# Hemodialysis Internal jugular vein versus Subclavian vein Catheters: Complications, patients’ comfort, tolerance and cost-effectiveness

**DOI:** 10.12669/pjms.35.1.249

**Published:** 2019

**Authors:** Muhammad Nadeem Shafique, Syed Hassan Akhtar, Miss Mahnoor, Mujahid Hussain

**Affiliations:** 1 Dr. Muhammad Nadeem Shafique, MBBS, MS Urology Assistant Professor, Sialkot Medical College, Sialkot, Pakistan; 2 Dr. Syed Hassan Akhtar, MBBS, FCPS Urology Assistant Professor, Kh Safdar Medical College, Sialkot, Pakistan; 3 Miss Mahnoor, Student MBBS (4^th^year) Federal Medical and Dental College, Islamabad, Pakistan; 4 Dr. Mujahid Hussain, PhD. Department of Biology, FG College, Sialkot Cantt, Pakistan

**Keywords:** Hemodialysis, Internal jugular vein, Subclavian vein, Catheter, Patient comfort

## Abstract

**Objective:**

To compare hemodialysis (HD) internal jugular vein (IJV) versus subclavian vein (SCV) catheters in terms of procedural complications, patients’ comfort, tolerance and cost effectiveness.

**Methods:**

Sixty six consecutive eligible adult patients planned for hemodialysis @ 3 sessions/ week for maximum 42 days in a private hospital at Sialkot, Pakistan were documented between March 2017 and April 2018. A group, IJV or SCV catheter was allotted to alternate subjects. The catheters were inserted as per practice guidelines. Record of catheter-related complications (CRCs) was computerized. Similarly, patients’ uncomfortability and expenditures on management of CRCs were recorded.

**Results:**

Of 66 cases, 62 (93.9%, 31/group) successfully completed the study. Baseline information showed male predominance (n = 47, 75.8%), age (*M* = 47, range 24-75 years) or catheter stay time (*M* = 40 days). The rate of vein damage or artery puncture was found higher in IJV than SCV group [(13.9 vs. 6.5%) or (9.7 vs. 3.2%), respectively] during catheterization. The difference also existed in late CRCs such as bacterial infection (32.3 vs. 16.1%), or device dysfunctioning (9.7 vs. 3.2%) with an exception of mechanical kinking. All the patients of IJV or SCV group with missed (19.4 vs. 6.5%) or shortened HD sessions (22.6 vs. 12.9%) reported CRCs-based discomfort as a cause of the regularity. Moreover, the participants of IJV group consumed 69% of the total expenditures on CRCs management.

**Conclusion:**

SCV is a better site for HD catheterization as it has comparatively lesser likelihood of complications, patients’ feel comfortable and it is also cost-effective.than IJV.

## INTRODUCTION

A remarkable reduction in the excretory function of the human kidney can be observed on chronic kidney disease^1^ (CKD) also called renal failure. Consequently, waste substances accumulate in the plasma and cause certain complications like pulmonary edema- the predictor for premature death. Unfortunately, Pakistan is a country where mortality rate on kidney diseases (majorly CKD) has touched a frightening figure of 13.5/100, 000 population.^2^

Hemodialysis (HD) is recommended as renal replacement therapy to sustain the life of the sufferer with low grade morbidity. Insertion of readily operational percutaneous double-lumen tunneled cuffed central venous catheter (CVC) at suitable vessel e.g. internal jugular^3^ or subclavian vein is the 1^st^ step towards efficient extracorporeal blood flow for hemofiltration. Similarly, the device acts as a bridge while switching from one type of permanent vascular access e.g. arteriovenous (AV) fistula to other type ‘AV graft^4^’on dysfunctioning.

The experienced practitioner inserts the device carefully using National guidelines and modern technologies. However, likelihood of short or long term CVC-related complications still exists at any of the three stages *viz*. insertion, stay in period, and removal (rarely). Arterial puncture or catheter-site bacterial infection usually emerges just after catheter’s placement; hence termed as early complications. However, central venous stenosis,^5^,^6^ thrombosis, mechanical kinking, or acute sepsis is observed as late catheter adverse outcome. The fear of the complications urges a patient to miss/shorten^7^,^8^ the HD sessions i.e. non compliance with therapy. The non-adherence to therapy results in poor hemofiltration - a challenge for the HD handlers. Moreover, magnitude of the dissatisfaction on treatment increases when sufferer (or even public health sector^9^) have to meet extra financial burden against CRCs’ management.

Open-accessed literature^6^,^10^ (with reference to Pakistan) is available on comparison between HD internal jugular vein (IJV) and subclavian vein (SCV) catherterization. However, authors of present study noticed scarcity on three areas *viz*. CVC-related complications (especially late), noncompliance with therapy due to patients’ feeling uncomfortable, or cost-effectiveness. To fill the gap, present work was planned. The aim of the study was to compare the outcomes of the HD catheterization at IJV and SCV in terms of the 3 areas. The findings will be useful for the professionals in general practice.

## METHODS

This experimental cross sectional study was conducted between March 2017 and April 2018 in a setting ‘The Kidney Centre’, Sialkot, Pakistan.

### Sampling of Subjects

Consecutive patients (aged>18 years) of either sex who were recommended for urgent hemodialysis (HD), shifted from peritoneal dialysis to HD, or needed change in position of the catheter on dysfunctioning of the previous vascular access were included. However, patients with missing previous medical record, reporting prophylactic administration of antibiotics, renal carcinoma, hemodialysis frequency (<3> per week), or severe psychological/mental issues were excluded. Group IJV or SCV catheter was assigned to alternate subjects (n = 66 i.e. 33 cases/group) from computer-generated list.

### Catheter Insertion

Pre-assessment and management of the subjects was conducted by physical examination along with laboratory work for urea/creatinine, complete blood count, serum electrolytes; ultrasonography and ECG. The raised K^+^ level was managed by inj. Calcium-Sandoz diluted I/V, inj. Sodabicarb 100 ml and 25% D/W 10 ml (2 ampoules) plus 6 units plain insulin I/V. Blind (i.e. without ultrasound guide) aseptic catheterization was commenced at locally anaesthetized site in vein of interest following Practice Guidelines^11^ for CV line. Moreover, issues of orthopnea were strictly addressed. The temporary subcutaneous double-lumen tunneled cuffed device remained intact for maximum six weeks i.e. till functioning of the permanent arteriovenous (AV) fistula/graft.

### Assessments

Baseline information of the subjects e.g. duration of dialysis were recorded. The infections were identified before classification vide codes of ICD-9-CM^12^ (*International Classification of Diseases, Ninth Revision, Clinical Modification*). The non-adherents were asked to give response (yes/no) against a question “Do CRCs cause non-adherence?” to assess the device-related uncomfortability. In each study group, the financial expenditures on management of the complications were pooled before %age estimation for cost-effectiveness.

### Ethical Considerations

The study was conducted after getting approval from the hospital ethics committee. Moreover, participation consent was mandatory for participants.

### Statistical Analysis

Data of age (continuous variable) was processed for mean+/-SD (range) values using statistical tool in SPSS ver. 16.0 (SPSS Inc., Chicago, IL, USA; Windows 2007). Non-adjusted odd ratio (OR) were calculated for different complications to visualize the comparative efficiency of the catheters.

## RESULTS

**Fig.1:**
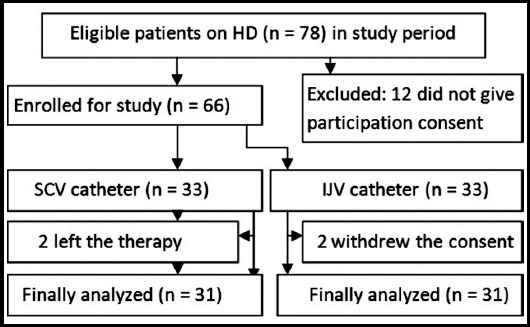
Flow sheet of subject sampling for site of HD catheters.

Of 66 cases, 62 (93.9%) showed adherence with prescription of clinicians for hemodialysis as shown in flow sheet of subject sampling (Fig.1). Males and females were in the ratio of 3.1:1 (47 vs. 15) with mean age of 47 (*SD* = 14, range 24-75) years. More than 50% population was on urgent HD. However, others switched from peritoneal dialysis to HD, or on replacement of dysfunctional central venous access i.e. AV fistula/graft. On the average, the patients held the temporary subcutaneous double-lumen tunneled cuffed device for 40 days.[Fig.1]

The heterogeneous, statistically insignificant data of early catheter-related problems is shown in Table-I. The rate of vein damage during placement of IJV catheter was higher than SCV catheter (13.9 vs. 6.5%). Similarly, likelihood of accidental artery rupturing was approximately three times more after IJV catheterization (95%CI: .316 – 32.741). However, no difference in cases was observed with reference to pulmonary complications (e.g. pleural rupture) or catheter site bacterial infections. [Table-I].

**Table-I:** Hemodialysis catheter-related early complications.

Variable	Population		OR (95% CI)

	SCV catheter	IJV catheter	
Vein damage; % *(f)*	6.5 (2)	13.9 (4)	2.15 (.364 – 12.693)
Artery rupture	3.2 (1)	9.7 (3)	3.21 (.316 – 32.741)
Pulmonary complications	3.2 (1)	3.2 (1)	1.0 (.060 – 16.737)
Bacterial infection*	6.5 (2)	6.5 (2)	1.0 (.132 – 7.587)

* exit-site infection; *p >*.05 (after chi-squared/Fisher’s exact test) against all variables.

Data in Table-II reveal late catheter-related complications observed during a period between catheter insertion and removals i.e. stay in time. The higher rate of stenosis (25.8%) was found in the patients of IJV catheter compared to matching SCV catheter group. Moreover, 3.21 times more dysfunctioning of IJV catheter (95% CI: .316 – 32.741) was noticed on account of thrombus formation or stenosis. In both the groups, the infections were identified as exit site or tunneled infections. Luckily, severe blood stream infection (BSI) was not reported in any case.[Table-II]

**Table-II:** Hemodialysis catheter-related late complications.

Variable	Population		OR (95% CI)

	SCV catheter	IJV catheter	
Device dysfunctioning; % *(f)*	3.2 (1)	9.7 (3)	3.21 (.316 – 32.741)
Thrombus formation*	3.2 (1)	3.2 (1)	1.0 (.060 – 16.737)
Central vein stenosis	19.4 (6)	25.8 (8)	1.45(.436 – 4.814)
Bacterial infection	16.1 (5)	32.3 (10)	2.48 (.733 – 8.369)
Infection-based replacement	12.9 (4)	25.8 (8)	2.35 (.645 – 8.814)

* managed through catheter removal and systemic anticoagulation

Comparatively higher numbers of patients from IJV group showed non adherence to the therapy through missing (n = 6, 19.6%) or shortening (n = 7, 12.9%) of HD sessions than SCV group (Table-III). All such subjects gave positive response (i.e. yes) against a question “Do CRCs cause the non-adherence?” However, almost same frequency of patients (reporting uneasiness in handling of device in daily life) was seen in both sides. The only plus point in the IJV side was lesser rate of mechanical kinking of the device compared to SCV (3.2 vs. 12.9%).

**Table-III:** Hemodialysis catheter-related discomfort in patients.

Variable	Population		OR (95% CI)

	SCV catheter	IJV catheter	
Missing of dialysis sessions; % *(f)* (Noncompliance with dialysis)	6.5 (2)	19.4 (6)	3.48 (.644 – 18.850)
Shortening of dialysis sessions (Noncompliance with dialysis)	12.9 (4)	22.6 (7)	1.969 (.512 – 7.563)
Uneasiness* in daily life	6.5 (2)	9.7 (3)	1.554 (.241 –10.010)
Kinking of device	12.9 (4)	3.2 (1)	0.225 (.024 – 2.139)

* due to handling

Evidently higher percentage i.e. 69% of the total financial burden on management of the CRCs was recorded against IJV group. In this group, the data of frequency of CRCs [No. of patients] was found, as: 1[9], 2[8], 3[4], 4[2], and 0 [8].

## DISCUSSION

Unpleasant experience of HD^13^ urges a sufferer to decline any research-oriented activity unless otherwise confidence building is ensured. Similarly, chance to leave the activity, as observed in present study can be expected from a hopeless person.

Dominance of male population (47 out of 62) is in good agreement to demographic pattern of previously published data^3^,^5^,^6^,^14^ on HD patients. The sex-specific differences in HD prevalence may be interpreted in terms of physiological differentiation, and social pros and cons about HD facility. Similarly, fair correspondence in age variable (*M* = 47, range 24-75) years and an Indian study^15^ advocates the commonalities in life style especially health care issues and aptitude towards regular checkup. The catheters remained intact for 40 days (on the average) i.e. 14 days more than reported (26 days) by Subramayam and Vakrani^9^ on similar cases. The reason behind prolongation would be some lacunae in underlying procedures and/or related complications.

Survival of the patient lies in successful HD for patients awaiting/not awaiting kidney transplant.^16^ Complication-free insertion of CVC for HD increases confidence of the patient on the professional’s competency even after switching from peritoneal dialysis^17^ on worst outcome. However, likelihood of vascular perforation^18^,^19^ by experience clinicians is a matter of great concern. Higher rate of damage to IJV than SCV in catheter placement procedure shows resemblance with a published data^3^,^20^ on same lines. Here, form/structure of the catheter is responsible for perforation ruling out any mistake by experienced clinician(s).

Catheter dysfunctioning is referred to failure in extracorporeal blood flow (Qb) of 300 mL/minutes. The reasons behind the problem include mechanical issues and/or thrombosis.^21^ More incidence towards IJV catheter of our study (in comparison to published data^3^,^10^,^19^) indicates sole mechanical issues. Catheter-related thrombosis (CRT)^4^ is actually thrombocytopenia and deserves management, accordingly. Higher incidence rate of the central vein stenosis^20^ is expected in the patients with inserted catheters especially SCV^6^ for hemodialysis as it is a commonly observed short-term complication. Double the rate of infection including bacteremia and tunnel infection on temporary IJV catheter marks the disadvantage of using this modality. Moreover, statistically insignificant difference in occurrence of the infection is in accordance with the findings of Zafarghandi et al.^19^ on IJV and SCV catheterization. An integrated protocol is used to address the infections especially catheter site infections,^4^,^22^ catheter-related bloodstream infections (CRBSIs)^3^,^10^,^23^,^24^ and dysfunctional device for standard outcomes.

Procedural complications and physical inconvenience in handling the catheter leads to missed and shortened dialysis sessions i.e. noncompliance/non-adherence to the scheduled RTT. The irregularities exert pressure on the clinicians to reduce the pathetic morbidity^7^ and risk of mortality. Surprisingly, IJV seems to pose such perception in relatively more numbers of the sufferers. The catheter needs proper care/handling of the catheter in dialysis^5^ and daily life. So, some handlers feel trouble in maintaining it. Mechanical kinking or disfiguring^4^,^10^,^25^ of the catheter is reset by the surgeon. However, in most of the cases its replacement is the only remedy.^26^ The kinking-base malfunctioning of SCV device might be the resultant of patient’s carelessness and catheter’s configuration.

The catheter-related complications (CRCs) e.g. malfunctioning of the device^5^, infections, and stenosis pose extra financial burden on the sufferer of hemodialysis and public health sector while dealing a costly hemodialysis^1^,^26^ to sustain the life of critical patients. More economic burden on the handlers of IJV device on change in vascular access or prior to utility of expensive AV fistula/graft^4^ is taken negative by helpless^12^ patients.

## CONCLUSION

Subclavian vein is a better site for HD catheterization as it involves comparatively lesser likelihood of device-related complications, patients’ feeling uncomfortable and cost-effectiveness than internal jugular vein. The patients’ compliance with renal transplant therapy is comfort dependent. However, patient’s consent should also be respected while deciding the placement site. Randomized trials on mega level are needed before rational decision-making approach about suitable place for the device insertion.

### Authors’ Contribution

**MNS:** Conceived, designed, collected data, financing, and manuscript’s final editing.

**HA and MM:** Collected data, and supported in manuscript writing.

**MH:** Applied statistical analyses, edited (language) and reviewed the manuscript.

## References

[1] Hill NR, Fatoba ST, Oke JL, Hirst JA, O'Callaghan CA, Lasserson DS (2016). Global prevalence of chronic kidney disease - A systematic review and meta-analysis. PLoS ONE.

[2] World Health Organization (WHO) (2017). World Health Report. Kidney disease; Pakistan. Geneva, Switzerland [Internet].

[3] Ruesch S, Walder B, Tramer MR (2002). Complications of central venous catheters:Internal jugular versus subclavian access - A systematic review. Crit Care Med.

[4] Bream PR (2016). Update on insertion and complications of central venous catheters for hemodialysis. Semin Intervent Radiol.

[5] Wang K, Wang P, Liang X, Lu X, Liu Z (2015). Epidemiology of hemodialysis catheter complications:a survey of 865 dialysis patients from 14 hemodialysis centers in Hennan, Province in China. BMJ Open.

[6] Afzal A, Haq AU, Ahmed A, Baig W, Zafar A, Ayub F (2017). Hemodialysis patients;Central vein stenosis following temporary double lumen catheterization in internal jugular and subclavian veins. Professional Med J.

[7] Obialo CI, Hunt WC, Bashir K, Zager PG (2012). Relationship of missed and shortened hemodialysis treatments to hospitalization and mortality:observations from a US dialysis network. Clin Kidney J.

[8] Shah R, Bhatt UY, Cleef SV, Farley M, Davis A, Swope M (2011). Vascular access thrombosis and interventions in patients missing hemodialysis sessions. Clin Nephrol.

[9] Subramayam TN, Vakrani GP (2018). Hemodialysis catheter related blood stream infections. Int J Res Med Sci.

[10] Khalid M, Malik MA, Bhatti MI, Gurmani MA, Jaskani IK (2017). Comparison of internal jugular vein with subclavian vein hemodialysis catheters access. J Uni Med Dent Coll.

[11] The American Society of Anesthesiologists (2012). Practice guidelines for central venous access;A report by the American Society of Anesthesiologists Task Force on central venous access. Anesthesiol.

[12] World Health Organization (2008). International Classification of Diseases. Clinical Modification (ICD-9-CM).

[13] Saeedi M, Ghafarzadeghan R, Hekmatpou D (2017). Perception of illness in patients undergoing hemodialysis:A qualitative study. Iran J Nursing.

[14] Hecking M, Bieber BA, Ethier J, Kautzky-Willer A, Sundaer-Plassmann G, Saemann MD (2014). Sex-specific differences in hemodialysis prevalence and practices and the male-to-female mortality rate:The Dialysis Outcomes and Practice Patterns Study (DOPPS). PLoS Med.

[15] Chaudhari ST, Sadavarte AV, Chafekar D (2017). Clinical profile of end stage renal disease in patients undergoing hemodialysis. MVP J Med Sci.

[16] Kaballo MA, Canney M, O'Kelly P, Williams Y, O'Seaghdha CM, Conlon PJ (2018). A comparative analysis of survival of patients on dialysis and after kidney transplantation. Clin Kidney J.

[17] Pajek J, Hutchison AJ, Bhutani S, Brenchley PEC, Hurst H, Perme MP (2014). Outcome of peritoneal dialysis patients and switching to hemodialysis:A competing risks analysis. Perit Dial Int.

[18] Sahutoglu C, Pestilci Z, Kocabas S, Askar FZ, Sunal SO, Cevik AG (2014). A venous catheter complication:Venous perforation and lung injury. Turk J Anaesth Reanim.

[19] Zafarghandi MR, Nazari I, Taghavi M, Salimi J, Moini M, Askarpour S (2013). Comparison of results of placement of cuffed -tunneled hemodialysis catheter in internal jugular vein with subclavian vein for long-term dialysis. Pol Przegl Chir.

[20] Agarwal AK, Patel BM, Haddad NJ (2007). Central vein stenosis:a nephrologist's perspective. Semin Dial.

[21] Marques MG, Maia P, Ponce P (2017). Dialysis catheter malfunction. Port J Nephrol Hypert.

[22] Qureshi AL, Abid K (2010). Frequency of catheter related infections in hemodialysed uraemic patients. J Pak Med Assoc.

[23] Raheem A, Rana AI, Mehmood SN, Ramzan M, Shah RA, Naseem S (2014). Two years experience with tunneled dialysis catheters in patients requiring hemodialysis. J Pak Med Assoc.

[24] Sabir O, Tarif N, Rizwan S, Rafique K, Rizvi N, Khan A (2015). Taurolidine lock to prevent catheter-related blood stream infections. Prof Med J.

[25] Vezzani A, Manca T, Vercelli A, Braghieri A, magnacavallo A (2013). Ultrasonography as a guide during vascular access procedure and in the diagnosis of complications. J Ultrasound.

[26] Miller LM, MacRae JM, Kiaii M, Clark E, Dipchand C, Kappel J (2016). Hemodialysis Tunneled Catheter Noninfectious Complications. Can J Kidney Health Dis.

